# Relationship of Malocclusions with Disorders of the Temporomandibular Joint in Children of CALI—Colombia

**DOI:** 10.1055/s-0041-1739450

**Published:** 2022-01-11

**Authors:** Nataly Mora-Zuluaga, Libia Soto-Llanos, Natalia Aragón, Katherine Torres-Trujillo

**Affiliations:** 1 Department of Pediatric Dentistry and Maxillary Orthopedics, School of Dentistry, University of Valle, Cali, Colombia; 2 Private Practice, Cali, Colombia

**Keywords:** temporomandibular joint, malocclusion, dentition, adolescents, children

## Abstract

**Objective** The aim of this study was to determine the relationship of malocclusion with the presence and severity of temporomandibular disorders (TMDs) in children.

**Materials and Methods** A clinical examination was performed in 87 patients (from 4 to 14 years of age) who attended the dentistry clinics of Universidad del Valle.

**Results** The 77 patients studied had malocclusions; 55 patients had TMD and 67.3% were female. The most frequent symptom of TMD was articular unilateral noise with 33.8%, followed by pain in at least one masticatory muscle with 26%. TMJ pain was observed in 24.7% of the patients. There was a statistically significant relationship between the presence and severity of TMD with type of dentition and transverse malocclusion, respectively.

**Conclusion** The presence of TMD in children with malocclusion presented in a high frequency. TMD depends on the type of dentition and its severity is dependent on transverse malocclusion.

## Introduction

The temporomandibular joint (TMJ) is a ginglymoarthrodial synovial joint that along with its groups of muscles can perform backward, forward, and bilateral mandibular movements. 1 The function of the TMJ allows articular movements in the three dimensions of space. 2 When their synergism is altered, 3 there is some TMJ dysfunction or disorder that can cause changes in everyday life. 4

Temporomandibular joint disorders (TMDs) have been associated with craniofacial abnormalities, malocclusions, and/or excessive overloads. 5 It is linked to progressive deterioration of bodily functions with age. 6 Its prevalence ranges from 20 to 50% in adults and 16 to 68% in children and adolescents. 4 7

There are signs and symptoms such as the presence of orofacial pain, limitation of the oral opening, TMJ pair or clicking sounds, among others reflect some alteration of the TMJ. 8 9 10 11 12 Dental changes and malocclusion are considered risk factors for the development of TMD, 13 with evidence of their relationship in 78 to 90% of cases. 8

The position of the condyle in glenoid fossa can predispose to the development of malocclusions because it influences the vertical, sagittal, and transverse relationships of the mandible, being important to maintain temporomandibular harmony with the dentition and to achieve stability of occlusion after maxillary orthopaedic treatment. 14 15 16 17

The classification of the malocclusion determines the patient's condition and the treatment plan. 18 19 20 The Helkimo index (HI) measures the severity and pain in patients with TMD taking in consideration both the anamnesis and the clinical and occlusal evaluation. The assessment of the modified HI parameters allows a quantification of the signs, symptoms and severity of the disorder. 21 Mandibular movements of pediatric patients are important to identify signs of TMD and they should be evaluated in relation to their age, sex, and type of dentition. 22 23 There is limited information on the relationship between malocclusions and TMD in children and adolescents. The aim of this study is to determine the relationship of malocclusion with the presence and severity of temporomandibular disorders (TMDs) in children.

## Materials and Methods

A descriptive cross-sectional study was performed. In it, 87 patients out of the 439 who attended the Pediatric Dentistry clinics of the Faculty of Dentistry of the Universidad del Valle (Cali, Colombia) between May 2018 and April 2019 were clinically evaluated. The sample was calculated by the difference of proportions of 25% in the group of exposed with an estimated *α* error of 5 and 80% power. Patients between 4 and 14 years of age, who presented some type of malocclusion in any of the three spatial planes and who had the approval of the parents to participate in the study through informed consent (Institutional Review Committee of Human Ethics 020–17) were included; patients with a systemic syndrome or disease, and/or who had undergone maxillary orthopaedic treatment or had had it, were not included.

**Fig. 1:**
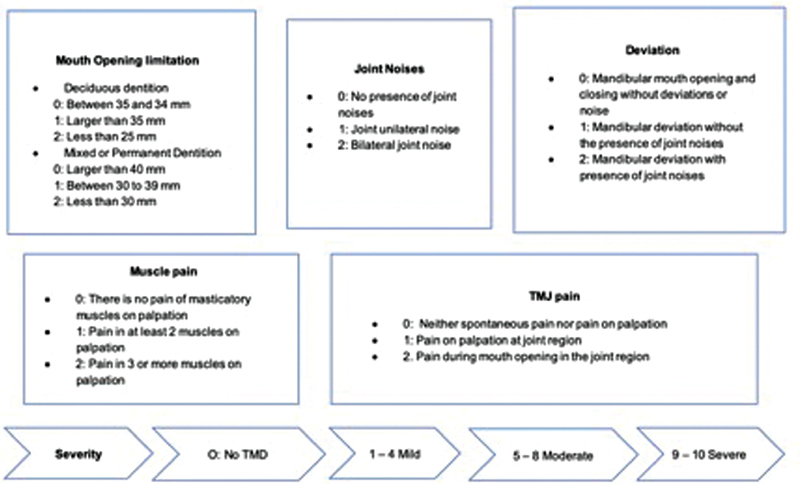
Evaluation score of the Helkimo Index (adjusted for evaluation in children). TMD, temporomandibular disorders.

The intraoral clinical examination was performed by researchers to determine the classification of malocclusions in the three planes of space: sagittal (Class I, Class II/1, Class II/2, Class III), vertical (normal, edge to edge, open bite, deep bite), transverse (posterior crossbite, scissor bite, normal), and presence of anterior crossbite, 20 24 25 type of dentition and other variables. To determine the presence and severity of TMD, the HI was used with some modifications. 5 11 21 The limitation of oral opening was evaluated by incisal references; joint noises were verified by palpation before opening and closing movements; mandibular deviation pattern was observed extraorally during opening and closing movements; muscle pain was evaluated by palpating along the muscle for one to two seconds in a resting position and joint pain due to bimanual pressure in the region with a closed and opened mouth. The score of each of the items in the modified HI was evaluated as shown in Fig. 1 .

The evaluators obtained an intra-interobserver Kappa index with a “very good” level that was above 0.81 in both cases. For data collection, a Google Form (Google tool) was used as an instrument that generated a dataset according to each variable. Each patient was assigned an alphanumeric code consistent with the order of admission. After the collection of the data from the 87 patients, the information was exported to Microsoft Excel.

The researchers performed a univariate analysis of the data through frequency tables and graphs using Microsoft Excel, the bivariate analysis that was used to measure the relationship between the response variable and each one of the independent variables were performed through Pearson's Chi-square statistical test with the STATA software.

## Results

Finally, 10 patients who did not present any type of malocclusion were excluded from the study, leaving a total of 77 children, with a median of age 7.42 years for males and 8.04 years for females. The type of dentition most frequent was mixed (71.4%) followed by the temporary (20.8%).

Class II division 1 sagittal malocclusion was characteristic of the studied population, followed by class I malocclusion. In vertical malocclusions, a deep bite was observed in a higher proportion in males (46.4%), while in females it was observed normal bite. In the transverse plane, the posterior crossbite was the most frequent malocclusion (16.3%) for females, while there was only one case in males. The anterior crossbite had a frequency of 9.1% in the general population and the sex distribution of the malocclusion is shown in Table 1 .

**Table 1:** Distribution *n* (%) of the population according to the type of malocclusion and sex

Variables		*n* (%)	
		Males	Females
Malocclusion			
Sagittal	Class I	15 (53.6)	15 (30.6)
	Class II/1	8 (28.6)	25 (51.0)
	Class II/2	1 (3.5)	2 (4.1)
	Class III	4 (14.3)	7 (14.3)
Vertical	Normal	9 (32.2)	26 (53.1)
	Border to border	3 (10.7)	3 (6.1)
	Deep bite	13 (46.4)	12 (24.4)
	Open bite	3 (10.7)	8 (16.4)
Transverse	Posterior crossbite	1 (3.6)	8 (16.3)
	Scissor bite	1 (3.6)	0 (0.0)
	Normal	26 (92.8)	41 (83.7)
Anterior crossbite	Presents:	3 (10.7)	4 (8.2)
	Does not present	25 (89.3)	45 (91.8)
Total		28 (100)	49 (100)

Note: School of Dentistry, 2019.

**Fig. 2:**
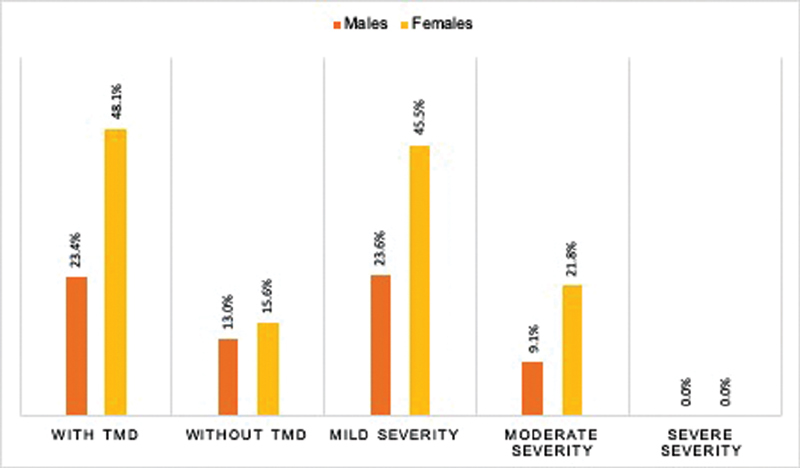
Distribution *n* (%) of the population according to sex and presence of temporomandibular disorders (TMD) and its severity. School of Dentistry, 2019.

The 71.5% of the patients presented at least one sign or symptom at the time of the clinical evaluation, being more frequent in females. The severity of TMDs was mostly in the mild category (69.1%) followed by a moderate category ( Fig. 2 ). The common symptom of TMD was articular unilateral noise (33.8%), followed by pain on palpation of at least one masticatory muscle (26%) and in the TMJ (24.7%). Painful symptoms occurred mainly in females and in the temporalis muscle, on the dominant side (data not shown).

In the sagittal plane, patients with class I and class II division 1 malocclusion, presented higher painful symptoms in at least one masticatory muscle and pain in the TMJ area; meanwhile, class III patients, presented greater deviation in mouth opening with articular clicking noises compared with the other groups. Only two patients class II division 1 and class III malocclusion had previous headache and preauricular pain. In the anterior crossbite category, the majority of patients presented mandibular deviation with articular bilateral clicking noise in mouth opening. Prior to the evaluation, deep bite patients presented headache and preauricular pain. During the evaluation they presented TMJ and masticatory muscles pain, and deviation in the mouth opening with a predominance of articular unilateral clicking noise. Lastly, in the transverse malocclusions, the patient with a scissor-bite had all the signs and symptoms, except pain in the TMJ region; and patients with posterior crossbite reflected unilateral clicking noise with TMJ symptoms on palpation ( Table 2 ).

**Table 2:** *n* (%) distribution of sagittal, ACB ^+^ , vertical and transverse malocclusion with the presence of signs and symptoms of TMD—Dentistry School, 2019

Variables		Sagittal malocclusion					Vertical malocclusion				Transverse malocclusion		
		Class I *n* = 30	Class II/1 *n* = 33	Class II/2 *n* = 3	Class III *n* = 11	ACB ^+^ *n* = 7	Normal bite *n* = 35	Deep bite *n* = 25	Open bite *n* = 11	Border to border bite *n* = 6	Normal bite *n* = 67	Posterior crossbite *n* = 9	Scissor bite *n* = 1
Buccal a opening	0	17 (56.6)	22 (66.6)	1 (33.3)	9 (81.8)	6 (85.7)	23 (65.7)	15 (60.0)	6 (54.5)	5 (83.3)	42 (62.6)	6 (66.6)	1 (100.0)
	1	11 (36.6)	11 (33.3)	2 (66.6)	1 (9.0)	0 (0.0)	10 (28.5)	9 (36.0)	5 (45.4)	1 (16.6)	23 (34.3)	2 (22.2)	0 (0.0)
	2	2 (6.6)	0 (0.0)	0 (0.0)	1 (9.0)	1 (14.2)	2 (5.7)	1 (4.0)	0 (0.0)	0 (0.0)	2 (2.9)	1 (11.1)	0 (0.0)
Deviation a	0	20 (66.6)	13 (39.3)	0 (0.0)	3 (27.2)	1 (14.2)	13 (37.1)	14 (56.0)	6 (54.5)	3 (50.0)	36 (53.7)	0 (0.0)	0 (0.0)
	1	3 (10.0)	8 (24.2)	1 (33.3)	1 (9.0)	1 (14.2)	7 (20.0)	4 (16.0)	1 (9.0)	1 (16.6)	11 (16.4)	2 (22.2)	0 (0.0)
	2	7 (23.3)	12 (36.3)	2 (66.6)	7 (63.6)	5 (71.4)	15 (42.8)	7 (28.0)	4 (36.3)	2 (33.3)	20 (29.8)	7 (77.7)	1 (100.0)
Joint noises a	0	20 (66.6)	19 (57.5)	0 (0.0)	4 (36.3)	2 (28.5)	17 (48.5)	17 (68.0)	6 (54.5)	3 (50.0)	43 (64.1)	0 (0.0)	0 (0.0)
	1	6 (20.0)	13 (39.3)	2 (66.6)	5 (45.4)	2 (28.5)	1 (2.8)	7 (28.0)	4 (36.3)	2 (33.3)	18 (26.8)	8 (88.8)	0 (0.0)
	2	4 (13.3)	1 (3.0)	1 (33.3)	2 (18.1)	3 (42.8)	5 (14.2)	1 (4.0)	1 (9.0)	1 (16.6)	6 (8.9)	1 (11.1)	1 (100.0)
Muscle pain a	0	18 (60.0)	23 (69.7)	1 (33.3)	6 (54.5)	0 (0.0)	27 (77.1)	11 (44.0)	7 (63.6)	3 (50.0)	43 (64.1)	5 (55.5)	0 (0.0)
	1	9 (30.0)	5 (15.1)	2 (66.6)	4 (36.3)	5 (71.4)	5 (14.2)	10 (40.0)	2 (18.1)	3 (50.0)	15 (22.3)	4 (44.4)	1 (100.0)
	2	3 (10.0)	5 (15.1)	0 (0.0)	1 (9.0)	2 (28.5)	3 (8.5)	4 (16.0)	2 (18.1)	0 (0.0)	9 (13.4)	0 (0.0)	0 (0.0)
TMJ a	0	22 (73.3)	23 (69.7)	1 (33.3)	7 (63.6)	4 (57.1)	22 (62.8)	19 (76.0)	9 (81.8)	3 (50.0)	49 (73.1)	3 (33.3)	1 (100.0)
	1	7 (23.3)	7 (21.2)	2 (66.6)	3 (27.2)	3 (42.8)	10 (28.5)	4 (16.0)	2 (18.1)	3 (50.0)	14 (20.9)	5 (55.5)	0 (0.0)
	2	1 (3.3)	3 (9.0)	0 (0.0)	1 (9.0)	0 (0.0)	3 (8.5)	2 (8.0)	0 (0.0)	0 (0.0)	4 (5.9)	1 (11.1)	0 (0.0)
Headache	Yes	2 (6.6)	2 (6.0)	0 (0.0)	2 (18.1)	2 (28.5)	1 (2.8)	3 (12.0)	1 (9.0)	1 (16.6)	4 (5.9)	1 (11.1)	1 (100.0)
	No	28 (93.3)	31 (93.9)	3 (100.0)	9 (81.8)	5 (71.4)	34 (97.1)	22 (88.0)	10 (90.9)	5 (83.3)	63 (94.0)	8 (88.8)	0 (0.0)
Preauricular pain	Yes	2 (6.6)	2 (6.0)	1 (33.3)	2 (18.1)	1 (14.2)	3 (8.5)	3 (12.0)	1 (9.0)	0 (0.0)	1 (1.4)	5 (55.5)	1 (100.0)
	No	28 (93.3)	31 (93.9)	2 (66.6)	9 (81.8)	6 (85.7)	32 (91.4)	22 (88.0)	10 (90.9)	6 (100.0)	66 (98.5)	4 (44.4)	0 (0.0)
Headache and preauricular pain	Yes	0 (0.0)	1 (3.0)	0 (0.0)	1 (9.0)	1 (14.2)	0 (0.0)	3 (8.0)	0 (0.0)	0 (0.0)	0 (0.0)	1 (11.1)	1 (100.0)
	No	30 (100.0)	32 (96.9)	3 (100.0)	10 (90.9)	6 (85.7)	35 (100.0)	23 (92.0)	11 (100.0)	0 (100.0)	67 (100.0)	8 (88.8)	0 (0.0)

Abbreviations: ACB + , anterior crossbite; TMD, temporomandibular disorders.

a Variables used to measure the presence and severity of TMD.

When performing the bivariate statistical analysis of malocclusions with the presence of TMD, a relationship was found with the type of dentition ( *p* = 0.031). In addition, there was statistical significance in the relationship between severity and transversal malocclusion ( *p* = 0.016). No statistically significant differences were found in the rest of the variables ( Table 3 ).

**Table 3:** Bivariate analysis of the presence and severity of TMD with sex, type of dentition, and malocclusions School of Dentistry, 2019

Variables	Presence of TMD		Severity of TMD	
	OR	*p-* Value	OR	*p-* Value
Sex	1.1000	0.294	1.2169	0.544
Dentition type	6.9300	0.031 a	8.0581	0.089
Sagittal malocclusion	4.0048	0.261	8.8029	0.185
Vertical malocclusion	0.6499	0.885	2.3509	0.085
Transverse malocclusion	4.597	0.1	12.1385	0.016 a
Anterior crossbite	2.6028	0.107	4.1576	0.125

Abbreviations: OR, odds ratio; TMD, temporomandibular disorders.

a Pearson's Chi-square test ( *p* <0.05).

## Discussion

Differences in the prevalence of TMD are attributed to the age range and the evaluation method used to determine its diagnosis, since there is no consensus on the diagnostic criteria or the use of validated instruments for this analysis in children and adolescents. 26

For the diagnosis of TMD, different parameters are considered such as: HI, Diagnostic criteria index for TMDs, craniomandibular index, and anamnestic questionnaires 27 28 29 30 ; these are only validated in the adult population. In this study, the HI was used with modifications of Thilander et al and Maglione et al, 5 11 based on physiological and clinical parameters of the child population. Therefore, it is confirmed that adequate and standardized methods are needed to identify the presence of TMD, understand, and approach pathological aspects opportunely, in this population. 31

It is observed that the severity of the disorder is related to the number of malocclusions present; patients with moderate severity had at least two types of malocclusions; These findings are similar to another study that reported that when the number of malocclusions increased, the clinical severity of the TMD also increased. 32

A study that evaluated patients in a similar age range, found the presence of at least one or more clinical signs of TMD in 25%, with a mild severity associated with the presence of posterior crossbite, anterior open bite, Class III malocclusion, and increased overjet. 5 The present study found a high frequency of TMD in patients with posterior crossbite, followed by anterior crossbite and open bite, but the most severe sagittal malocclusion was Class II division 1 malocclusion.

Another similar study estimated, a mild severity of 13.6% and moderate of 2.8%, being greater in females, and a total presence of TMD of 40%, related to more signs present in patients with sagittal malocclusion class III, followed by patients with class II division 1 13 ; results differ with the present study as there was a greater presence of TMD (71.5%) and a greater relationship in patients with class II division 1 malocclusion. On the other hand, a more recent study shows a frequency of TMD of 72.3%, and even reports a presence of mandibular deviation during mouth opening (53%), 33 higher than that estimated in Cali-Colombia.

The relationship between the anterior crossbite with the manifestation of muscle pain was established in another investigation, such as the relationship between the unilateral posterior crossbite with the presence of TMD. 28 These results are similar to the estimates presented in this research, where it was determined that the unilateral posterior crossbite is the most frequent malocclusion when there is greater severity of TMD.

Eighty percent of the patients with mixed dentition had a TMD sign or symptom, 36.4% articular noise; and muscle pain 23.4 and 14.3% in women and men, respectively. In contrast, Mexico reports a prevalence of TMD of 20.7% in mixed dentition and similarity, reporting muscle pain more frequent in the female population and articular noises (35%). 34

In Colombia, a population with mixed dentition reported the prevalence of signs and symptoms associated with TMD, greater in females; however, this study did not take into account the presence of malocclusions. 35 Another Colombian population evaluated with an anamnestic questionnaire and a clinical examination found that 36% of the children had signs and symptoms associated with TMD; the most frequent sign was positive palpation in the masticatory muscles (32%), predominating the masseter muscles, followed by articular noises (10%). 9 These results differ from the present study, reporting a higher prevalence of TMD, muscle pain (37.7%) with no significant difference between muscle groups and articular noises.

A study in Asian adolescents reports the presence of TMD in 61.4%; and additionally evaluated other related factors (not taken into account in this study) such as: depression, stress or sleep disturbances (33%) and anxiety (65.2%), which was significantly related to TMD, 36 37 which favors when considering the management of associated factors during the pathology treatment plan. 38

### Clinical Significance

This study contributes to the scientific and clinical evidence, expanding knowledge in the pediatric dentistry field since the presence of signs and symptoms of TMDs in children and adolescents is increasingly frequent and this type of pathologies has been mainly related to the adult population.

## Conclusion

The presence of TMD signs and symptoms in children with malocclusions occurs with high frequency when performing the corresponding clinical examination. It depends on the type of dentition. mixed dentition behaves as a risk factor and severity dependent on transverse malocclusion, the posterior crossbite being the most related. Most of the studies were performed especially in the adult population and reports on the child population are still lacking. The multiple existing indices for the evaluation of TMD make it difficult to accurately compare with the results found in different publications, however the use of the RDC/TMD protocol is recommended for future research. It is important that dentists and specialists are aware of routine joint area exploration to determine the presence of TMD in children, as TMD is increasing in this population.
